# Cardioembolic Stroke Diagnosis Using Blood Biomarkers

**DOI:** 10.2174/1573403X10666140214122633

**Published:** 2013-11

**Authors:** VÍctor Llombart, Teresa GarcÍa-Berrocoso, Alejandro Bustamante, Israel Fernández-Cadenas, Joan Montaner

**Affiliations:** 1 Neurovascular Research Laboratory, Institut de Recerca Vall d’Hebron. Barcelona, Spain. Neurovascular Unit. Department of Neurology. Universitat Autònoma de Barcelona. Hospital Vall d’Hebron. Barcelona. Spain;; 2 Fundació per la Docència i Recerca MÚtuaTerrassa, Terrassa. Barcelona, Spain

**Keywords:** Atrial, biomarker, cardioembolic, classification, etiology, fibrillation, stroke, miRNA.

## Abstract

Stroke is one of the main causes of death and disability in the world. Cardioembolic etiology accounts for approximately one fifth of all ischemic strokes whereas 25-30% remains undetermined even after an advanced diagnostic workup. Despite there is not any biomarker currently approved to distinguish cardioembolic stroke among other etiologies in clinical practice the use of biomarkers represents a promising valuable complement to determine stroke etiology reducing the number of cryptogenic strokes and aiding in the prescription of the most appropriated primary and secondary treatments in order to minimize therapeutic risks and to avoid recurrences. In this review we present an update about specific cardioembolic stroke-related biomarkers at a protein, transcriptomic and genetic level. Finally, we also focused on reported biomarkers associated with atrial fibrillation (a cardiac illness strongly related with cardioembolic stroke subtype) thus with a potential to become biomarkers to detect cardioembolic stroke in the future.

## INTRODUCTION

Stroke remains one of the most important neurological affection representing the second leading cause of preventable death and being one of the major causes of productivity impairment. In the US, on average, every 40 seconds, someone has a stroke and annually, 5.5 million people die worldwide, with 44 million disability-adjusted life-years lost [1, 2]. Furthermore, the prevalence of stroke is expected to become significantly larger as the world population older than 65 is increasing by 9 million people per year [3]. 

There exist two principle acute stroke subtypes. The main one, representing over 80-85% of all strokes, is ischemic stroke caused by brain arterial occlusion. On the other hand, 15-20% are due to bleeding into the brain as a consequence of an arterial rupture [1]. At present, stroke diagnosis is mainly based on clinical criteria supplemented by imaging data. In most countries, once diagnosed patients with acute ischemic stroke are treated with intravenous recombinant tissue plasminogen activator (r-tPA), a serine protease, as primary therapy. Unfortunately, with a narrow effective therapeutic time window of only 4.5 h, an early diagnostic becomes essential. Moreover, after overcoming diagnosis and hyperacute treatment, an accurate etiological classification is critical not only during the acute phase and its primary therapy, but also and mainly to select a suitable secondary treatment. 

## MAJOR STROKE ETIOLOGY CLASSIFICATION SYSTEMS

Since the Harvard Stroke Regisitry development in 1978 [4], several classifications of stroke subtypes have been developed, being the TOAST (Trial of Org 10172 in Acute Stroke Treatment) classification system the most widely used nowadays for stroke subtyping. The TOAST classification system determines five major subtypes of ischemic stroke: cardioembolic stroke (CE), large artery atherotrombotic stroke (LAA), small vessel disease (SVD), stroke of other determined cause (SOC) and stroke of undetermined cause [5]. As well as TOAST, all the other classification systems group patients into different main categories being, in some cases, further sub-classified in other subtypes. Using some of the currently available stroke classification systems there is an evident oversizing and a great heterogeneity of the undetermined category, especially in the eldest ones. This might probably be a direct consequence of the unavailability of modern diagnostic tools by the time that these classification systems were developed, hindering a complete evaluation of the patient [4-6]. 

In contrast, new classifications such as SSS-TOAST, its automated version CCS (Causative Classification of Stroke System) [7, 8], the Korean TOAST [9] and ASCO (Atherosclerosis, Small-vessel disease, Cardiac source, Other cause) [10, 11], have been developed taking into account underlying diseases associated with stroke and the existence of different phenotypes. As a consequence, the rate of strokes with an undetermined cause has been clearly reduced with the newest stroke classification systems when compared with the eldest ones.

The latest classification system, CISS (Chinese Ischemic Stroke Subtype Subclassification), is a two step system which was conceived in 2011. CISS first step aims at the etiology, considering five TOAST-based categories but including more accurate subgroups, whereas the second step classifies stroke patients by the mechanism that underlies the ischemic event [12]. Even though considering both etiological and pathophysiological causes of stroke, one of the most important limiting conditions of the CISS classification system is its dependence on modern imaging technology availability. 

## STROKE MANAGEMENT REGARDING ETIOLOGICAL SUBTYPES

Depending on the causes of the artery occlusion patients might be differently managed in the stroke unit. If the thrombus has a cardiac or atherosclerotic origin, those affected individuals who fulfil the inclusion criteria receive r-tPA as a primary treatment. On the contrary there exists controversy about thrombolysis on patients with lacunar strokes. Some authors are against the administration of intravenous r-tPA in patients without demonstrated artery occlusion [13], whereas others propose thrombolysis even on the presence of an undetectable fibrin clot in the arteriole [14, 15]. In addition, as hemorrhagic transformation risk is related to the volume of the infarcted tissue [16], that risk might be reduced in lacunar strokes. Nevertheless the degree to which thrombolytic primary therapy may improve outcome in these patients is still uncertain [17].

Regarding secondary prevention therapy, patients will receive different treatment depending on etiology: cardioembolic strokes are usually treated with anticoagulant drugs whereas atherosclerotic strokes follow an antiplatelet therapy or even surgery (e.g. carotid endarterectomy). Similarly antiplatelet agents are the treatment of choice for lacunar strokes, together with antihypertensive drugs. 

In spite of the importance of the accurate stroke subtype classification, the etiology of approximately 30% of all stroke cases remains unknown even after a precise and advanced diagnostic workup has been conducted [18]. The use of biomarkers would ease the lowering of the rate of cryptogenic strokes and also could contribute to speed up the diagnostic process prescribing the most appropriated primary and secondary treatments in order to minimize therapeutic risks and to avoid recurrences.

## BIOMARKERS OF CARDIOEMBOLIC STROKE 

Stroke caused by cardiac embolism accounts for approximately one fifth of ischemic strokes each year and 6-12% patients experience recurrences within 2 weeks after the first embolism [19]. Thrombus formation in the cardiac chambers is mainly caused by blood stasis, leading to a fibrin rich clot which may be then ejected towards the arterial circulation. Cerebral ischemia appears when the blood flow through a cerebral artery turns impaired by this thrombus. 

One of the purposes of the stroke etiological classification systems is to classify patients for the therapeutic decision-making but, in spite of their usefulness, these classification systems still have important limitations (i.e. high rates of undetermined etiology, neuroimaging dependence). Biomarkers of stroke etiology might have a great importance in the development of more precise and reliable classification schemes which may serve as a valuable research and diagnostic tool [20].

This review presents an update of the research done in stroke biomarkers focusing on the possibility of identifying stroke of cardioembolic etiology since a more aggressive management will reduce dramatically stroke recurrence rates. For that we performed a search in Medline database introducing the terms *cardioembolic stroke AND biomarker* and selected data on different available biomarkers that may be measured in blood, such as proteins and circulating microRNAs, or analyzed at genetic level as gene expression profile or polymorphisms.

**Fig. (1):**
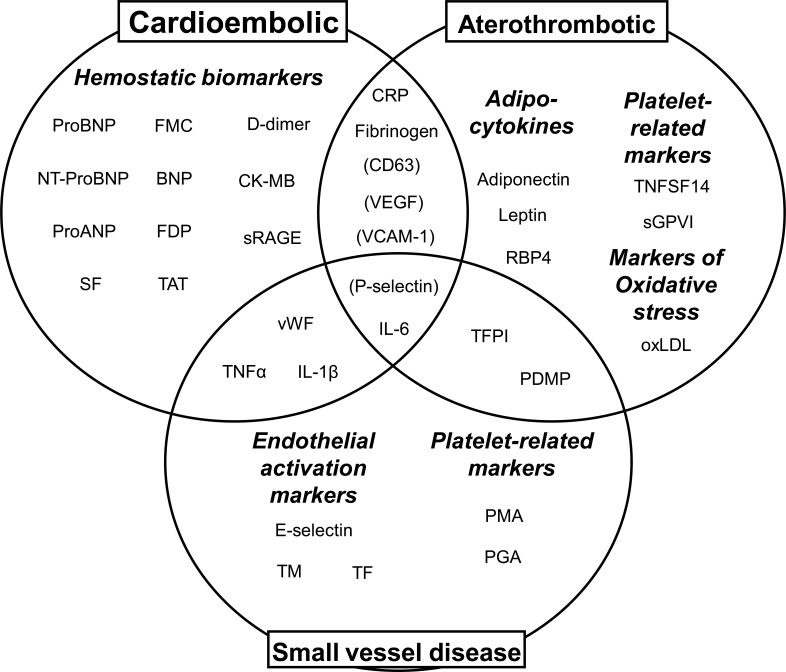
Most relevant biomarkers grouped by specific pathophysiologies related to the three main stroke etiologies. Molecules in brackets have been identified in patients with AF, strongly related with cardioembolic stroke. BNP: b-type natriuretic peptide; CK-MB: creatine kinase isoform MB; CRP: C reactive protein; FDP: fibrin/fibrinogen degradation products; FMC: fibrin monomer complex; IL-1β: interleukin 1 beta; IL-6: interleukin 6; oxLDL: oxidized low denisity lipoprotein; PDMP: plateled derived microparticles; ProANP: pro-atrial natriuretic peptide; RBP4: retinol binding protein; SF: soluble fibrin; sGPVI: soluble glycoprotein VI; sRAGE: soluble advanced glycation end products receptor ; TAT: thrombin-antithrombin complex; TFPI: tissue factor pathway inhibitor; TM: thrombomodulin; TNFα: tumour necrosis factor alpha; TNFSF14: tumour necrosis factor family member 14; vWF: von Willebrand factor.

The most promising candidates to become biological markers for each stroke etiology are supposed to play a role in the pathophysiology of each stroke subtype (i. e. hemostasis, inflammation, immune system activation, endothelial damage or oxidative stress) [21]. Unfortunately several of these processes underlie in more than one stroke etiology, highlighting the complexity of the cerebrovascular disease. What is more, biomarkers might not even be specific to stroke since other clinical conditions, such as myocardial infarction, have common underlying pathophysiological mechanisms. Thus non-specific biomarkers should be employed with caution and this may hamper the application of these biomarkers in daily clinical practice. The most relevant studied biomarkers and their association with stroke etiologies are summarized in (Fig. **1**).

## STROKE ETIOLOGY AND CIRCULATING PROTEINS 

**Fig. (2):**
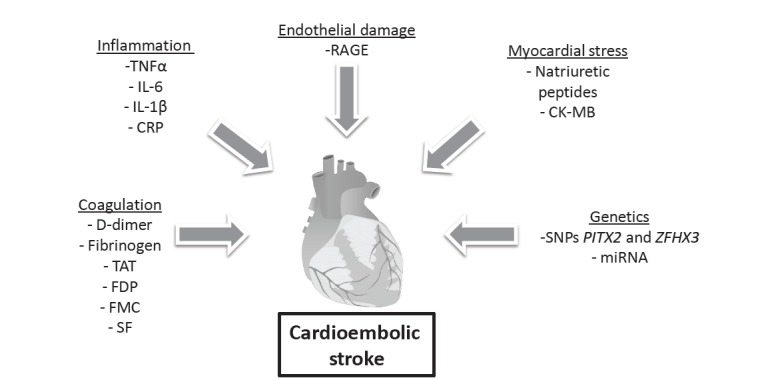
Illustration of the cardioembolic stroke pathophysiology and different areas represented by the biomarkers presented in the review.

In the subsections below we will focus on protein biomarkers associated with cardioembolic stroke grouped by the underlying pathophysiological mechanism. The biomarkers discussed are represented in (Fig. **2**) and classified by the pathophysiologic mechanism they are involved in. 

### Coagulation and Fibrinolytic Systems

Coagulation and fibrinolytic mechanisms are activated as a physiologic response after ischemia. D-dimer, a breakdown product of fibrin, is one of the basic markers of fibrinolytic system activity. It is not only elevated in plasma taken from patients with atrial fibrillation (AF) -most common cardiac abnormality leading to stroke- but also has been found raised in CE stroke patients compared with other etiologies [22-24]. A recent study, conducted by Alvarez-Perez and collaborators with 200 stroke patients and 50 controls, shows a thrombogenic profile in patients with CE stroke characterized by higher D-dimer levels than LAA, SVD or undetermined etiologies (p<0.0001) [25]. 

Raised fibrinogen and D-dimer/fibrinogen ratio in patients with CE stroke (p<0.0001 vs controls and patients with LAA and SVD strokes; p=0.004 vs patients with undetermined stroke) were also found in this study. D-dimer together with other hemostatic biomarkers, such as thrombin-antithrombin III complex (TAT), fibrinogen, fibrin/fibrinogen degradation products (FDPs), fibrin monomer complex (FMC) and soluble fibrin (SF), have been assayed lately among different subtypes of stroke [26]. CE stroke patients showed higher levels of FMC, SF, D-dimer and FDP than non-CE patients (LAA, SVD and others, p<0.05) after one day of hospitalization while fibrinogen was increased in LAA strokes when compared to CE. FMC and SF levels (both markers indicative of hypercoagulable state) were still different (p<0.05) two days after stroke onset, but regarding FDP (indicative of hyperfibrinolytic state), these differences remained even 7 days after stroke. Thus, measurement of these three hemostatic biomarkers could aid in the differentiation of CE and non-CE strokes in both acute and subacute phases.

Other markers for CE stroke have been further investigated. B-type natriuretic peptide (BNP) has an antifibrotic role in the heart and acts as a cardiac hormone. BNP is the main product of the cleavage of propeptide (proBNP), which also equimolarly release N-terminal peptide (NT-proBNP). All three peptides are considered to have a similar potential to discern CE stroke subtype among other etiologies [27]. Montaner and colleagues showed an association between high levels of BNP and D-dimer with CE stroke [24]. Plasma concentrations of both proteins were determined in a cohort of 707 patients. BNP higher than 76 pg/ml (OR: 2.3; 95% CI: 1.4-3.7; p=0.001) together with D-dimer higher than 0.96 μg/ml (OR: 2.2; 95% CI: 2.4-18.9; p=0.001) were independent predictors of CE stroke in a logistic regression model including NIHSS at baseline. This is important since CE strokes present larger infarcts and more pronounced deficits than other etiologies. Thus logistic-regression models should be adjusted by variables which reflect this involvement such as NIHSS, infarct size or Oxford Community Stroke Project classification (OCSP). More recently published studies report similar relation between BNP and cardioembolism and further confirm previous findings [24, 28-30]. Interestingly, BNP has also been found to be associated with AF (OR: 2.0; 95% CI; 1.6-2.5) in a cohort of 569 patients including all stroke subtypes. In the same study BNP was related with CE stroke subtype (p<0.001) and resulted an independent predictor of functional outcome ((OR, 0.5; 95% CI, 0.3-0.9) and mortality (OR, 3.05; 95% CI, 1.1-8.2) exclusively in patients with CE [31].

NT-proBNP has also been proposed for discerning between CE and other stroke etiologies in 92 patients with AF [27]. As authors discuss, NT-proBNP serum levels above 912 pg/mL (sensitivity 55.5%, specificity 97.9%, positive predictive value (PPV) 90.9 %, negative predictive value (NPV) 83.9%) can be used to identify those patients more prone to have AF in undetermined strokes and consequently it may aid to select subjects for prolonged cardiac rhythm monitoring in order to confirm paroxysmal AF. These patients who would probably suffer a secondary stroke with a cardiac origin may benefit from anticoagulant preventive treatment.

Regarding proBNP, Rodríguez-Yáñez and colleagues published a study including 262 patients from all TOAST etiologies and showed that plasma levels over 360 pg/mL of this peptide were independently associated with CE stroke (OR: 28.51; 95% CI: 5.90-136.75; p<0.0001) in a model that included basal NIHSS [32].

Von Willebrand Factor (vWF), a well studied hemostatic marker, has been found to be related with CE stroke. Raised levels of vWF were determined in CE patients by Licata and collaborators but only showed a trend (10 (5-12) ng/ml; p=0.0053) compared to other stroke subtypes [33]. Other studies showed that high levels of vWF are associated with AF and can predict cardiovascular events [34-36]. More recently, Hanson and collaborators examined vWF levels in patients with CE, LAA SVD or UND stroke [37]. Subject with CE and LAA stroke displayed higher vWF levels than SVD group (253 IU/dl [95% CI 230-279] and 263 IU/dl [95% CI 242-286], vs. 213 UI/dl [95% CI 197-229], respectively) during the acute phase, whereas at three months after the cerebrovascular event, only patients who suffered CE stroke showed higher vWF than those with SVD (240 IU/dl [95% CI 224-256] vs. 201 IU/dl [95% CI 189-215]). As authors discussed, these results agree with those from Ohira and colleagues, in which CE stroke patients also showed increased levels of vWF than patients with SVD (p<0.05) [38].

Other biological markers of cardiac damage such as proatrial natriuretic peptide (pro-ANP) together with creatine kinase MB (CK-MB) were analyzed and reported to be higher in patients with CE infarcts. Authors suggested a cutoff point of 2.6 ng/mL (sensitivity 62%, specificity 80%, p<0.0001) and 2266.6 fmol/mL (sensitivity 62%, specificity 70%, p<0.0001), for each marker respectively [32].

Very recently Santamarina and collaborators determined plasma concentrations of some of these previously studied biomarkers (BNP, D-dimer, CK-MB, troponin and myoglobin) in a cohort of 89 selected patients with undetermined stroke. After cardiological work-up, 49 of these patients with cryptogenic stroke were diagnosed as embolic. The most common findings were AF, severe aortic atheromatosis, patent foramen ovale, aneurysm of atrial septum and dilated cardiomyopathy. Cardioembolic stroke patients had higher plasma concentrations of BNP (121 [24-260.5] vs 26.8 [12.2-70.4], p=0.003), myoglobin (109 [66.9-177.7] vs 85.4 [61.4-125], p=0.028) and CK-MB (1.45 [0.5-2.27] vs 0.5 [0.5-1.4], p=0.004) than the ones that remained as cryptogenic. Finally, they constructed a predictive model adjusted by age, gender, risk factors, heart disease and stroke severity where BNP and CK-MB together with suffering of a previous stroke were independently associated to embolic abnormalities (OR 8.06, CI 95% 2.34-27.72; OR 6.52, CI 95% 1.44-29.5; OR 5.34, CI 95% 1.14-29.97; respectively). These results evidence the fact that some biomarkers contain the potential to improve the diagnosis of a stroke from an embolic source optimizing the performance of early cardiac explorations in those patients with undetermined stroke [39].

### Systemic Inflammation and Endothelial Activation

High serum levels of immuno-inflammatory mediators such as TNFα, IL-6, IL-1β, selectins and adhesion molecules have been associated with ischemic stroke suggesting that both molecular and cellular inflammation mediates the mechanisms of cerebral injury and repair. Several studies have tried to clarify the role played by inflammation in the pathogenesis of different stroke etiologies. Licata and collaborators evaluated the levels of different cytokines, selectins and adhesion molecules among ischemic stroke subtypes [33]. In this study, patients classified as CE showed higher concentration of TNF-α (38.5 (22.2-46) pg/ml; p<0.0001), IL-6 (11 (5.5-19) pg/ml; p=0.0029) and IL-1β (11.5 (8-13) pg/ml; p<0.0001) in plasma compared to other TOAST categories. Although systemic inflammatory state have been further related with atherosclerosis, these results suggest that inflammation may also play a role in the pathological processes of cerebral cardioembolism [40]. Moreover, higher plasma levels of IL-18 [41], E-selectin [42], soluble vascular cell adhesion molecule-1 (sVCAM-I) [43], platelet factor-4 (PF-4) [44], sP-selectin [45], Platelet Microparticles (PMP) [46], asymmetric and symmetric dymethylarginine (ADMA, SDMA) [47, 48] found in different cohorts of patients with AF indicates that inflammation, platelet activation and oxidative stress are conditions not only related with LAA but might be also related with CE stroke.

C reactive protein (CRP) is a well-known acute phase reactant protein whose concentration rises in response to inflammation or infection. In spite of being one of the most studied inflammatory markers in stroke, several controversial results have been reported regarding its use as etiologic biomarker. Terruzzi and collaborators showed higher plasma levels of CRP in CE stroke patients when compared with other TOAST subtypes within the first 6 hours after symptoms onset [49]. However, as CRP has been suggested to promote platelet activation and foam cells generation through macrophages differentiation, it has been associated with large artery atherotrombotic events [50, 51]. Álvarez-Perez and colleagues showed a higher CRP in LAA when compared with SVD (p=0.010) and undetermined (p=0.003) strokes, but not with CE ones [52]. Adding more controversy to the role of CRP as etiologic biomarker, some authors detected no differences in CRP serum levels between etiology groups [53, 54]. Alternative approaches, such as meta-analysis or studies including greater cohorts of patients, are necessary to clarify the relation between CRP levels and different stroke etiologies.

### Endothelial Damage

Micro and macroangiopathy affect endothelial walls thickness and reduce blood flow through small and large vessels. These processes are strongly related with hypertension and diabetes.

Reduced plasma levels of soluble advanced glycation end products receptor (sRAGE), a cell surface molecule member of the immunoglobulin superfamily, are considered to be related with the development of microangiopathy. Plasma levels of this protein were compared between stroke etiologies in a total of 482 enrolled patients by Yokota and collaborators. Distribution of sRAGE were significantly different among etiologies, being CE plasma concentrations the highest (1280 (271-4720) pg/mL, p=0.001) [55]. In this line of evidence, Montaner also showed significantly higher sRAGE levels in the group of patients with CE stroke (1.1 (0.7-1.9) ng/ml) etiologic [24].

Biomarkers which have been assayed in ischemic stroke patients and related with CE subtype are summarized in Table **1**. Table **2** shows the CE stroke-related biomarkers with a highest sensitivity and specificity found in the literature.

## OTHER CANDIDATES

Apart from stroke biomarkers strongly related with CE etiology, some other molecules are associated with high risk cardioembolic conditions and therefore remain as candidates to become markers for CE stroke. The most common of these cardiac conditions which may lead to a cardioembolic event are: atrial fibrillation, recent myocardial infarction, dilated myocardiopathy, and mitral rheumatic stenosis. Other major sources of cardioembolism are ineffective endocarditis, marantic endocarditis and atrial myxoma; and patent foramen ovale, atrial septal aneurysm, atrial or ventricular septal defects, calcific aortic stenosis, and mitral annular calcification as minor sources [56, 57]. These potential biomarkers of underlying pathological risks may provide ample basis for future studies and could become valuable indicators of neurovascular events allowing physicians to carry out a better etiological classification. 

Table **3** shows those candidates which are associated to AF, the most common CE stroke related cardiac affection. 

## GENE EXPRESSION BIOMARKERS OF STROKE ETIOLOGY

Gene expression profile can also be used to discern between different stroke subtypes. In 2010, Jickling GC and colleagues reported the results of the complete CLEAR trial. The expression profile from a total number of 23 CE and 10 LAA patients were compared and, as a result, 40 genes whose expression was significantly different among both etiologies were identified [58]. 11 of these 40 genes are involved in cellular movement, cell-to-cell signalling and interaction, and tissue development. 

Especially interesting genes are *ENPP2 (Ectonucleotide pyrophoshpatase/phosphodiesterase family member 2)* and *GRM5 (Glutamate receptor metabotropic 5)*. ENPP2 protein is involved in angiogenesis and neurite growth, whereas *GRM5* encodes the glutamate receptor metabotropic 5, which is involved in normal brain function. Other genes such as *LHFP*, *TMEM19* and *EBF1* are involved in cardiac proliferation, cardiovascular development and haematological system function [77]. The proposed 40 genes expression profile predicted correctly 9 out of 10 patients known to be CE and when the same gene profile was used in 36 patients with an undetermined etiology, 58% of them were reclassified as CE or LAA with a probability greater than 90%. These results strongly suggest that gene expression profile could be used to complement diagnostic tests to determine the etiology in cryptogenic patients. 

On the other hand, in the same study a different 37 genes expression profile was found to distinguish patients with CE stroke due to AF and non-AF causes with a probability higher than 90%. This 37 genes list may provide physicians an additional tool for the identification of patients more prone to suffer AF, as its detection strongly depends on cardiac monitoring, not always available for all patients. Moreover, paroxysmal AF detection requires a more prolonged follow-up period making more difficult the diagnosis of AF, thus this 37 gen expression profile analysis at baseline could be very helpful to identify these individuals.

In a later study, the same authors followed a similar strategy in a cohort of patients with lacunar and non-lacunar stroke. The study reported 41 genes whose expression profile allowed to distinguish both groups and predict etiology in small deep infarcts of undetermined cause [78].

Finally, these two gene profiles, the 40-genes list that distinguished CE and LAA strokes and the 41-genes list that distinguished lacunar and non-lacunar strokes, were integrated and combined with infarct location assessed by neuroimaging to predict the most probable cause of stroke in a cohort of 131 patients with cryptogenic strokes. Of these 131 undetermined strokes, 76 (58%) were predicted to be CE, 24 (18,3%) were LAA, 15 (11,5%) were lacunar and 16 (12,2%) remained unknown [77]. 

As authors discussed, these studies have some limitations being the small population size the most important. However, they evidenced that gene expression profile could allow us to distinguish between different causes of stroke. With the development of point-of-care devices, based on fast analysis, cryptogenic stroke clarification and preventive stroke treatments could be further improved.

## STROKE ETIOLOGY AND CIRCULATING microRNA 

microRNAs (miRNAs) are small (19-25 nt length) non-coding RNAs involved in the post-transcriptional regulation of genes by inhibition or degradation of mRNA. miRNAs are single stranded molecules of RNA whose high stability allows its detection in serum or plasma. Moreover, their expression patterns may reflect underlying pathophysiological processes. These important features make miRNAs very attractive biomarkers for human diseases [79]. 

Unfortunately, just a few studies have attempted to find differential miRNA levels between patients and controls. The study conducted by Gan and colleagues is an example. They measured blood levels of miRNA-145 (a modulator of vascular smooth muscle cells phenotype) and showed an up-regulation in stroke [80]. On the other hand, Zeng analyzed blood miRNA-210 and found it decreased in patients with ischemic stroke when compared to healthy controls (p=0.001) even after 14 days from stroke onset [81]. 

Among etiologies, until now only one study has focused on finding differences on miRNAs expression [82]. This study, conducted by Tan and collaborators, assessed differential miRNA profile and reported a list of 132 miRNAs with altered expression depending on stroke etiology. Some of them were up-regulated with a fold change >2 in only one subtype. This is the case of 7 miRNAs (miR-130b, -29b, -301a, -339-5p, -532-5p, - 634 and 886-5p) more expressed in SVD. Moreover, cluster analysis on different miRNA profiles among etiologies showed that undetermined stroke profiles were similar to SVD, suggesting that studied patients with undetermined etiology have resulted from SVD. Hence it might be assumed that miRNA profiling could be used to differentiate CE, LAA or SVD strokes from each other and to resolve undetermined strokes.

In contrast to the small number of published studies of miRNA focused on stroke etiologies, numerous studies have documented a relation between some specific miRNA and AF, strongly related with CE stroke [83]. Very recently, Liu Z and collaborators examined miRNA expression profiles in 3 groups: 5 patients with paroxysmal AF, 5 with persistent AF and 5 healthy controls. They found substantially lower levels of miRNA-150 in both groups of AF patients when compared with controls (p<0.001) and was identified as a predictor of AF (OR 1.96, 95% CI 1.5 to 3.57, p<0.001) [84]. Other authors have evidenced the involvement of miRNAs in basic mechanisms of AF. Adam and colleagues found an increased expression on miRNA-21 and a decreased expression of protein Sprouty1 (downstream target of miRNA-21) in left atria of patients with AF [85]. They also showed a reduction of AF by miRNA-21 inhibition, evidencing its important role in atrial fibrosis regulation.

The results of these studies suggest, on one hand, the use of miRNA as plasma biomarkers for AF detection and consequently the identification of an embolic source in undetermined strokes; on the other hand, they open the door to potential therapeutic targets by direct miRNA silencing.

## STROKE ETIOLOGY AND GENE POLYMORPHISMS 

To date several studies have been performed to further understand the genetic basis of ischemic stroke. The most promising results have been obtained from projects that performed Genome Wide Analysis (GWA) approaches. The International Stroke Genetics Consortium (ISGC) and the Wellcome Trust Case Control Consortium 2 (WTCCC2) conducted a GWA study on three stroke subtypes: LAA, SVD and CE stroke [86]. This was a large study with a discovery association phase conducted in 3548 affected individuals and 5972 controls, and a second replication phase performed in 5859 cases and 6281 controls. Some previously reported associations were replicated in this analysis such as the single nucleotide polymorphisms (SNPs) rs1906599 and rs12932445, located in the *PITX2 *(Paired-like homeodomain 2) and *ZFHX3* (Zinc finger homeobox 3) genes respectively, and both related with CE etiology; or the SNP rs2383207 located in the *CDKN2A/CDKN2B *(Cyclin-dependent kinase inhibitor 2) gene, related with LAA stroke. Interestingly, a previously unknown association between LAA stroke and the SNP rs11984041 (located within the final intron of the *HDAC9 *(Histone deacetylase 9) gene) was firstly identified in this study. Finally, a third additional verification step was performed in 735 LAA cases and 28583 controls which further confirmed previous data. Taken together, the results from the discovery and two-steps verification phases provide strong evidence for association between the new SNP rs11984041 and LAA stroke (p=1.87x10^-11^) and further confirmed the association between CE stroke etiology and previously identified polymorphism in genes *PITX2* and *ZHF3*. 

Other studies have been carried out with approaches different from GWA, identifying other SNPs related to stroke etiology subtypes, e.g. rs2020918 related with SVD [87, 88], or rs315934, rs1180243, rs2071373 associated with a decrease in LAA stroke [89]. In spite of being size-limited and needing replication in further steps, these studies provide helpful information as a basis for future GWA of polymorphisms related to stroke etiologies. 

Other polymorphisms different from single nucleotide ones have been analyzed and observed to be specifically associated with a stroke subtype. For instance, a 32 base-pair deletion in *CCR5 *(Chemokine C-C motif receptor 5) gene (Δ32 polymorphism) is considered to exert a protective effect against CE stroke as it has lower frequency in this subtype (OR, 0.4; 95% CI, 0.24-0.79; p=0.008) than in LAA, SVD or cryptogenic strokes [90]. 

Although blood proteins, nucleic acids and even gene expression profiles may aid in the diagnosis and decision-making during acute and subacute phases of stroke, genetic biomarkers specific for each etiology provide valuable evidence before stroke occurrence allowing physicians to prescribe the most suitable preventive treatment. Furthermore, the identification of genetic signatures of each etiology may be of main interest in guiding undetermined stroke diagnosis, especially during subacute phase when it becomes primordial an optimal secondary prevention to minimize recurrences. Table **4** shows a compilation of CE stroke and AF related genes, miRNA and polymorphisms.

## FUTURE PERSPECTIVES

During the last years, a great number of studies have been conducted aiming to find new biomarkers that could aid in the differentiation of ischemic stroke etiologies. Although some of these studies have shown markers with high sensitivity and specificity, in a large cohort of patients and proving to be independent predictors of ischemic stroke, there are still some hurdles to take over before implementing them to daily clinical routine. Table **5** shows the general strengths and shortcomings of the studies on stroke etiology biomarkers. 

Recent advances achieved in proteomics in the last few years have brought new approaches for the identification of biomarkers. The use of quantitative proteomics has proven to be a promising strategy for identifying potential biomarkers for a number of diseases such as cancer, infectious diseases or autoimmunity. The stroke field can also take benefit from this fast development since recent studies have reported very promising results mainly directed at diagnosis. Dawson and collaborators explored the urinary proteome from ischemic stroke and transient ischemic attack patients versus controls. They developed two biomarker-based classifiers with identified proteins and validated them in independent blinded samples, showing a clear association of these biomarkers and stroke [91]. Dayon and colleagues also analyzed proteins from brain extracellular fluid and identified stroke-related proteins (i.e. glutathione S-transferase, or peroxiredoxin-I) that were further validated [92]. The same strategies could be followed on selected patients with clear etiologic profile to detect new candidate markers for each stroke subtype. Once replicated and validated, the combination of different biomarkers in a diagnostic test would provide the most valuable approach to accurately identify stroke etiology. However, neither biomarker nor panel of biomarkers has still showed enough sensitivity and specificity to improve clinical predictor models. New statistical tools, such as net reclassification improvement (NRI) and integrated discrimination improvement (IDI) indexes, have been developed to evaluate the added value of a biomarker or combination of biomarkers to the clinical basis [93, 94]. If biomarkers demonstrated its role, these improved prediction models would help to reclassify patients according with the risk of suffering a cardiac embolism. Thus the rate of undetermined strokes might be reduced and a faster decision-making in pharmacological secondary prevention could be done.

At a genetic level, recent studies in transcriptomic and specially those performed through GWA approaches have provided new stroke and even etiology-related markers. These newly described genetic markers may not only aid in the diagnosis or patient etiologic classification, but also may serve as future therapeutic targets for each stroke etiologic subtype.

Despite having found many encouraging candidates by all these approaches their clinical application requires several careful validation steps. Multi-centric studies, with greater cohorts of patients, supported by multiple replications and performed with tools for massive molecular and genetic analysis will be the base to increase the output of potential biomarkers that may be implemented in daily clinical practice.

## Figures and Tables

**Table 1.** Candidate makers of cardioembolic stroke.

Name	Type of Biomarker	Protein Accession #	Sample size	Protein Levels Regarding Etiology	Main Function	Ref
Albumin	Protein	P02768	200	CE < other etiologies	Regulates colloidal osmotic pressure of blood.	[52]
ANP	Protein	P01160	262	CE > other etiologies	Cardiovascular homeostatic hormone.	[32]
BNP	Protein fragment	-	1300	CE > other etiologies	ProBNP cleavage product. Cardiac hormone that regulates cardiovascular homeostasis.	[24, 29, 31, 39]
CK-MB	Protein	P12277-P06732**	262	CE > other etiologies	Mediates energy transduction in tissues. Increased in heart damage diseases.	[32, 39]
CRP*	Protein	P02741	985	CE > other etiologies; LAA > other etiologies; no difference among subtypes	Displays several functions associated with host defense. Risk marker of AF.	[49-54, 59]
D-dimer	Protein fragment	-	1430	CE > other etiologies	Fibrin degradation product.	[22-26, 52, 59, 60]
FDP	Group of proteins	-	69	CE > non-CE	Involved in coagulation/fibrinolysis processes.	[26]
Fibrinogen*	Protein	P02671	269	CE < LAA; no difference among subtypes	Yields monomers that polymerize into fibrin and is a cofactor in platelet aggregation.	[25, 26]
FMC	Protein aggregate	-	69	CE > non-CE	Involved in coagulation/fibrinolysis processes.	[26]
IL-1β	Protein	P01584	227	CE > other etiologies	Pro-inflammatory cytokine.	[33, 40]
IL-6	Protein	P05231	227	CE > other etiologies	Pro-inflammatory cytokine.	[33, 40]
NT-proBNP	Protein fragment	-	92	CE > non-CE	Pro-BNP cleavage product. High levels may help to select stroke patients with AF.	[27]
Pro-BNP	Protein	P16860	262	CE > other etiologies	BNP precursor. ProBNP might be useful to reclassify undetermined stroke as of CE origin.	[32]
S100B	Protein	P04271	33	CE > other etiologies	Involved in metal-ion binding and in the regulation of protein phosphorylation in brain tissue.	[61]
sCD40L*	Protein	P29965	132	No difference among etiologies	Inflammatory marker. Elevated in AF patients.	[62, 63]
SF	Protein complex	-	69	CE > other etiologies	Involved in coagulation/fibrinolysis processes.	[26]
sRAGE	Protein	Q9UQ07	1189	CE > other etiologies	Growth factor for several cell types.	[24,55]
TNF-α	Protein	P01375	227	CE > other etiologies	Pro-inflammatory cytokine.	[33,40]
vWF	Protein	P04275	1551	CE and LAA > other etiologies	Promotes platelet adhesion to the sub-endothelial matrix. Higher in patients with AF.	[33-38]

ANP: atrial natriuretic peptide; AF: atrial fibrillation; BNP: b-type natriuretic peptide; CE: cardioembolic; CK-MB: creatine kinase MB; CRP: C-reactive protein; FDP: fibrin/ fibrinogen degradation products, FMC: fibrin monomer complex; IL-1β: interleukin 1-beta; IL-6: interleukin 6; LAA: large artery atherosclerosis; ; NT-proBNP: N-terminal part of pro-BNP; Pro-BNP: proform of BNP; SF: soluble fibrin; sRAGE: soluble receptor for advanced glycation end products; SVD: small vessel disease; TNF-α: tumour necrosis factor alpha;

* Candidate marker with controversial results among studies.

** UniProt codes from CK-MM and CK-BB.

**Table 2.** Markers with highest sensitivity / specificity for the identification of CE stroke.

Marker	Sample Size	Cutoff Point	Sensitivity	Specificity	PPV	NPV	Ref
BNP	707	>76 pg/mL	68%	72%	55%	82%	[24]
D-dimer	707	>300 ug/L	100%	52%	46%	73%	[24]
BNP combined with D-dimer	707	-	66.5%	91.3%	-	-	[24]
Pro-BNP	262	>360 pg/mL	87%	83%	-	-	[32]
ANP	262	>2266.6 fmol/mL	62%	70%	-	-	[32]
NT-proBNP	92	>265 pg/mL	71.4%	73.7%	77.8%	66.6%	[27]
CK-MB	89	>1.5 ng/mL	47.9%	85%	79.3%	79.3%	[39]
BNP combined with CK-MB	89	-	31.2%	95%	88.2%	53.5%	[39]

ANP: atrial natriuretic peptide; BNP: b-type natriuretic peptide; CK-MB: creatine kinase MB; NT-proBNP: N-terminal part of pro-BNP; NPV: Negative predictive value; PPV: Positive predictive value

**Table 3.** Candidate markers related with atrial fibrillation.

Name	Protein Accession #	Sample Size	Biomarker Levels in AF	Main Function	Ref
Adiponectin	Q15848	384	Persistent AF > paroxysmal AF and control subjects	Adipokine involved in the control of fat metabolism and insulin sensitivity.	[64, 65]
ADMA	-	42	Acute AF > chronic AF and controls	Endogen inhibitor of NOS. ADMA contributes to thromboembolism in AF.	[47]
Ang-2	Q15123	59	AF > control subjects	Angiogenic factor.	[66]
Apelin	Q9ULZ1	166	AF < sinus rhythm or control subjects	May have a role in the control of body fluid homeostasis.	[67,68]
CD63*	P08962	121	AF > control subjects	Platelet activation marker.	[69]
E-selectin*	P16581	145	AF > control subjects	Marker of endothelial activation.	[42]
Fibrinopeptide A	P02671	83	AF ↑	Released as part of blood clotting process. It reflects thrombin activity.	[59]
IL-18	Q14116	56	Persistent AF > paroxysmal AF and sinus rhythm	Pro-inflammatory cytokine.	[41]
MMP-1	P03956	48	AF < control subjects	Cleaves collagen types I, II and III.	[70]
MMP-2	P08253	364	AF > sinus rhythm	Degrades extracellular matrix in remodeling of vasculature, angiogenesis and tissue repair.	[43,71]
MMP-3	P08254	86	AF > sinus rhythm	Degrades fibronectin, laminin, gelatins and collagens.	[71]
MMP-9	P14780	364	AF > control and sinus rhythm subjects	Proteolyses extracellular matrix.	[41,71,72]
NPY	P01303	222	AF > control subjects	Implicated in the control of feeding and in secretion of gonadotrophin-releasing hormone.	[72]
Osteoprotegerin	O00300	2863	Associated with AF	Neutralizes osteoclastogenesis.	[73]
PF-4	P02776	26	AF > control subjects	Marker of platelet activation in AF patients.	[44]
PMP	-	20	AF > control subjects	PMPs play a role in hemostatic response to vascular injury.	[46]
Prothrombin fragment 1.2 (F1+2)	-	48	AF > control subjects	Marker of thrombogenesis	[59]
p-selectin (CD62P)	P16109	121	AF > control subjects	Mediates the interaction of activated endothelial cells or platelets with leukocytes	[69]
SDMA	-	394	AF > non-AF	It influences NO formation via inhibition of L-arginine uptake.	[48]
sVCAM-1	P19320	278	Associated with AF	Important in cell-cell mediation.	[43]
sP-selectin	P16109	90	AF > control subjects	Mediates interaction of activated endothelial cells or platelets with leukocytes.	[45]
TGF-β	P01137	107	AF < non-AF	Controls proliferation, differentiation and other functions in many cell types.	[74]
TIMP-1	P01033	134	AF > sinus rhythm patients and control subjects	Irreversibly inactivates metalloproteinases.	[70, 71]
Troponin I	P48788	6189	AF ↑	Inhibitory subunit of troponin. Increased troponin I associated with an increase in the risk of stroke or systemic embolism and vascular events.	[75]
VEGF	P15692	72	Paroxysmal and persistent AF > control subjects	Pro-angiogenic factor.	[76]

Ang: Angiopoietin; ADMA: Asymmetric dimethylarginine; SDMA: Symmetric dimethylarginine: CITP: Carboxy-terminal telopeptide of collagen type I; MMP: Matrix metalloproteinase; TIMP-I: Tissue inhibitor of metalloproteinases; IL: Interleukin; NPY: Neuropeptide Y; PF-4: Platelet factor 4; PMP: Platelet microparticle; TF: Transferrin; TGF-β: Transforming growth factor beta; VEGF: Vascular endothelial growth factor; sVCAM-1: soluble vascular cell adhesion molecule 1; sCD40L: soluble CD40 ligand; NOS: Nitric oxide synthase.

* Candidate marker with controversial results among studies.

**Table 4.** Genes, miRNA and polymorphisms candidates to become biomarkers of CE stroke:

Genes associated with CE stroke				
Candidate Molecule	Entrez Gene	Sample Size	Reason for Being a Candidate	Ref
*ENPP2*	5168	33	Regulates lysophosphatidic acid production. It has Angiogenic properties.	[77]
*GRM5*	2915	33	Modulates normal brain function.	[77]
*LHFP*	10186	33	Involved in cardiac proliferation.	[77]
*TMEM19*	55256	33	Cardiovascular development and haematological system function.	[77]
*EBF1*	13591	33	Involved in haematological system development and function.	[77]
miRNA Associated with CE Stroke				
Candidate Molecule	Accession Number	Sample size	Reason for Being a Candidate	Ref
miRNA-145	MI0000461	32	Regulates the smooth muscle cells differentiation. Up-regulated in ischemic stroke patients when compared to controls.	[80]
miRNA-210	MI0000286	112	It is strongly linked to hypoxia pathways, and it is up-regulated in response to hypoxia-inducible factors. Decreased expression in ischemic stroke compared with control subjects.	[81]
miRNA associated with AF				
Candidate molecule	Accession number	Sample size	Reason for being a candidate	Ref
miRNA-150	MI0000479	10	Regulates hematopoiesis by modulating stem cell differentiation to megakaryocytes. Lower levels in AF patients than control subjects	[84]
miRNA-21	MI0000077	10	Regulates atrial fibrosis. Increased plasma levels than control subjects.	[85]
Polymorphisms Associated with CE stroke				
Candidate Gene	SNP	Sample Size	Reason for Being a Candidate	Ref
*PITX2*	rs1906599	3548	Controls cell proliferation in a tissue-specific manner and is involved in morphogenesis. SNP associated with CE stroke.	[86]
*ZFHX3*	rs12932445	3548	Transcriptional repressor. Regulator of myoblasts differentiation. SNP associated with CE stroke.	[86]
*CCR5*	-	478	δ32 polymorphism (32 bp deletion) associated with lower risk of CE stroke than LAA, SVD and cryptogenic subtypes.	[90]

* ENPP2: Ectonucleotide pyrophoshpatase/phosphodiesterase family member 2; GRM5: Glutamate receptor metabotropic 5; LHFP: lipoma HMGIC fusion partner; TMEM19: *transmembrane protein 19*; EBF1: *early B-cell factor 1*; PITX2: Paired-like homeodomain 2; ZFHX3: Zinc finger homeobox 3; CCR5: Chemokine C-C motif receptor 5; SNP: single nucleotide polymorphism; CE: cardioembolic; LAA: large artery aterothrombotic; SVD: small vessel disease.

**Table 5.** General pro’s and con’s of studies conducted aiming to find ischemic stroke etiology biomarkers.

Strenghts	Shortcommings
Some published studies include large cohorts (n>200 patients). The great majority of studied biomarkers for stroke etiology can be easily determined in blood samples. The most promising biomarkers are related to specific chronic condition prior to brain artery occlusion (i.e. cardiac dysfunctions or insufficiency). Some biomarkers, especially when combined, have showed high sensitivity and specificity for cardioembolic stroke i.e D-dimer & BNP combination [24], NT-proBNP [27], etc Some biomarkers have been proven to clear up undetermined strokes, i.e D-dimer & BNP combination [24] or a panel including 40 genes expression profile [77]. Most of those studies show optimal cutoff points for the studied biomarkers and perform logistic regression models to find out if a biomarker might independently predict cardioembolic stroke. Some of the markers may be determined some days after the acute event and still have an acceptable predictive value. Relevant clinical decisions may be done using these markers (i.e. to shift antiplatelet to anticoagulant regime).	There is currently no “gold-standard” to define the exact etiology/mechanism of stroke. Some underlying conditions i.e. inflammation, endothelial damage are common among different stroke etiologies, making some of the biomarkers unspecific. The clot formation usually occurs hours before sample collection. Therefore, a biomarker specifically related to this event might be partly cleared from circulation at that time. Most of times samples are taken after 4.5 hours, thus currently the great majority of the studied biomarkers might not be useful during acute phases. Most published studies do not report temporal profiles of the biomarker after stroke onset and among etiologies, therefore the ideal time point of the biomarker to be used remains elusive. Biomarker studies, especially those with small sample size, do not show a biomarker cutoff point and a logistic regression analysis nor other statistical metrics (i.e. IDI). Cardioembolic related biomarkers might be also associated with the cardiovascular disorders originating the index stroke. Ongoing therapies prior to stroke onset are not always taken into account in patient’s selection criteria. Previous anticoagulation or antiplatelet therapies might influence biomarker levels. Clinical models are not always adjusted by NIHSS and infarct size. As CE stroke is more pronounced than other etiologies it might be a confounder to be taken into account.
